# Dysregulation of the urea cycle – potential targets for treatment and diagnosis in prostate cancer

**DOI:** 10.1017/erm.2026.10059

**Published:** 2026-06-16

**Authors:** Michael Ladurner, Helmut Klocker, Hannes Neuwirt, Iris E. Eder

**Affiliations:** 1 Department of Urology, Medical University of Innsbruck, Innsbruck, Austria; 2 Department of Internal Medicine IV – Nephrology and Hypertension, Medical University of Innsbruck, Innsbruck, Austria

**Keywords:** arginine, biomarker, ornithine, polyamine synthesis, prostate cancer, therapy, urea, urea cycle

## Abstract

**Background:**

Prostate cancer (PCa) exhibits profound metabolic reprogramming, including dysregulation of the urea cycle (UC), an emerging driver of tumour growth, therapy resistance, and immune modulation. In normal physiology, the UC detoxifies ammonia via urea production. In PCa cells, however, altered expression of UC enzymes such as argininosuccinate synthase 1 (ASS1), arginase (ARG) and ornithine decarboxylase (ODC1) reroutes metabolites towards other pathways, including polyamine and nucleotide synthesis. These changes enhance proliferation, facilitate metabolic plasticity and influence the tumour microenvironment.

**Methods:**

This review summarises mechanistic insights into UC dysregulation in PCa, with emphasis on its interplay with the tricarboxylic acid (TCA) cycle, choline metabolism, and polyamine synthesis.

**Results:**

We critically evaluate current therapeutic strategies such as arginine deprivation (PEGylated arginine deiminase, ADI-PEG 20, PEGylated recombinant human arginase 1, PEG-BCT-100), ODC1 inhibition (α-difluoromethylornithine, DFMO), and glutaminase blockade (CB-839) and discuss their integration with other therapies, including androgen deprivation. Furthermore, we explore the challenges and the usefulness of UC metabolites as diagnostic and prognostic biomarkers for early detection of PCa as well as for a follow-up of patients during therapy.

**Conclusions:**

We conclude that UC dysregulation represents a targetable metabolic vulnerability in PCa. However, successful translation will require combinatorial therapeutic approaches and rigorous validation of UC-based biomarkers in prospective clinical trials.

## Introduction

Prostate cancer (PCa) is a biologically heterogeneous tumour of elderly men, which counts as the most common type of cancer among men in the European Union (Ref. 1) and the second most common cancer in men worldwide (Ref. 2), with inevitably rising incidence rates in the future due to the ageing population. The recent cancer statistics reports that PCa alone accounts for 30% of new cancer diagnoses per year in the United States (US) (Ref. 3). Besides age, family history and race are well-accepted risk factors for PCa (Ref. 4). Moreover, various epidemiological studies revealed that dietary habits, body mass index and lifestyle as well as metabolic disorders such as metabolic syndrome and diabetes have an impact on cancer development and progression (Ref. 5). When detected at an early localised stage, PCa can be effectively managed by active surveillance, surgical removal of the prostate or radiotherapy, depending on risk characteristics (Ref. 6). Advanced PCa, on the other hand, is usually treated by androgen deprivation therapy (ADT), which is highly effective per se in treatment-naïve patients but suffers from common progression to castration resistance. A number of novel drugs, which are either given alone or in combination, have been developed. This resulted in a significant overall improvement in the survival of patients and – importantly – in a complete change in the clinical management of patients with metastatic PCa (mPCa) (Ref. 7). Nevertheless, treatment resistances are a major problem in advanced stages and therefore mPCa unfortunately remains a lethal disease, actually ranging on the second position after lung cancer among all cancer deaths in the US (Ref. 3). Hence, early diagnosis together with novel strategies to overcome treatment resistances is highly important.

Metabolic reprogramming is a hallmark adaptation of cancer cells to enable energy production and synthesis of cell building blocks for enhanced growth and survival. It therefore represents a valuable and broad working surface to develop novel therapy approaches. In this review, we will focus on the role of the urea cycle (UC) in the metabolic dysregulation of PCa cells and discuss its potential use in the development of diagnostic markers and therapeutic drugs. We will summarise some of the most interesting approaches and also provide their actual status of research from preclinical to clinical testing.

## Metabolic reprogramming of PCa cells

**Figure 1.**
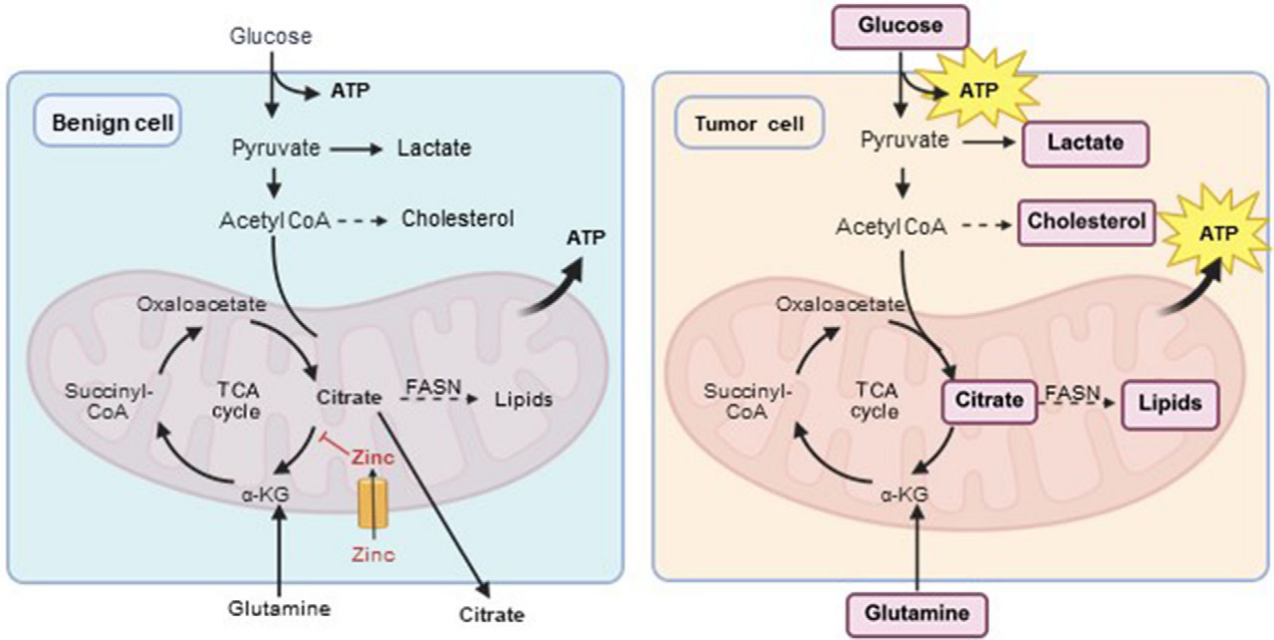
Tricarboxylic acid (TCA) cycle activity in benign and tumour cells of the prostate. Benign prostate cells accumulate high levels of zinc so that citrate is not metabolised via the TCA cycle but secreted into the seminal fluid instead. In tumour cells, by contrast, citrate becomes available for energy production through the TCA cycle and oxidative phosphorylation. At the same time, citrate becomes highly accessible for lipid synthesis with the key enzyme fatty acid synthase (FASN) and glucose is used for lactate and cholesterol synthesis. In tumour cells, glutamine is besides glucose the most important amino acid for energy production. Its metabolisation is catalysed via glutaminase (GLS). ATP: adenosine triphosphate; a-KG: alpha-ketoglutarate; CoA: coenzyme A; FASN: fatty acid synthase; GLS: glutaminase; TCA: tricarboxylic acid. This image was created in https://BioRender.com. Figure 1. long description.

The prostate has a very unique metabolic phenotype. Normal prostate epithelial cells accumulate high levels of zinc, which inhibit the activity of mitochondrial aconitase (ACO2) and thus prohibit citrate from entering the TCA cycle (Ref. 8) (Figure 1). This results in an unusually low TCA cycle and oxidative phosphorylation activity but high levels of citrate instead, which are secreted into the seminal fluid (Ref. 8). Besides citrate, high levels of spermine are accumulated and secreted by the normal prostate, indicating a high activity of polyamine biosynthesis (Ref. 9). PCa cells reprogram this ‘zinc-citrate regulation’ to re-activate the TCA cycle for higher energy production, most likely through reducing the expression of zinc transport proteins, which consequently results in lowering intracellular zinc concentrations (Ref. 10). PCa cells typically exhibit high *de novo* lipid synthesis activity and a preferential use of fatty acids for energy production (Ref. 11) together with enhanced oxidative phosphorylation activity (Ref. 12). In addition, high choline levels sustain membrane phospholipid production and cellular division (Ref. 13). Notably, the metabolic activity of PCa may change during therapy and disease progression. It has been shown, for instance, that tumour cells may shift from a lipogenic phenotype into a more glycolytic phenotype during tumour cell progression (Ref. 14), rendering glucose and lactate important energy substrates for advanced stages of PCa (Ref. 15). Various studies also reported on metabolic changes within the UC of PCa cells (Ref. 16) although the significance of the UC in PCa and its use in diagnosis and treatment has been somehow overshadowed.

## The urea cycle in benign prostate cells

**Figure 2.**
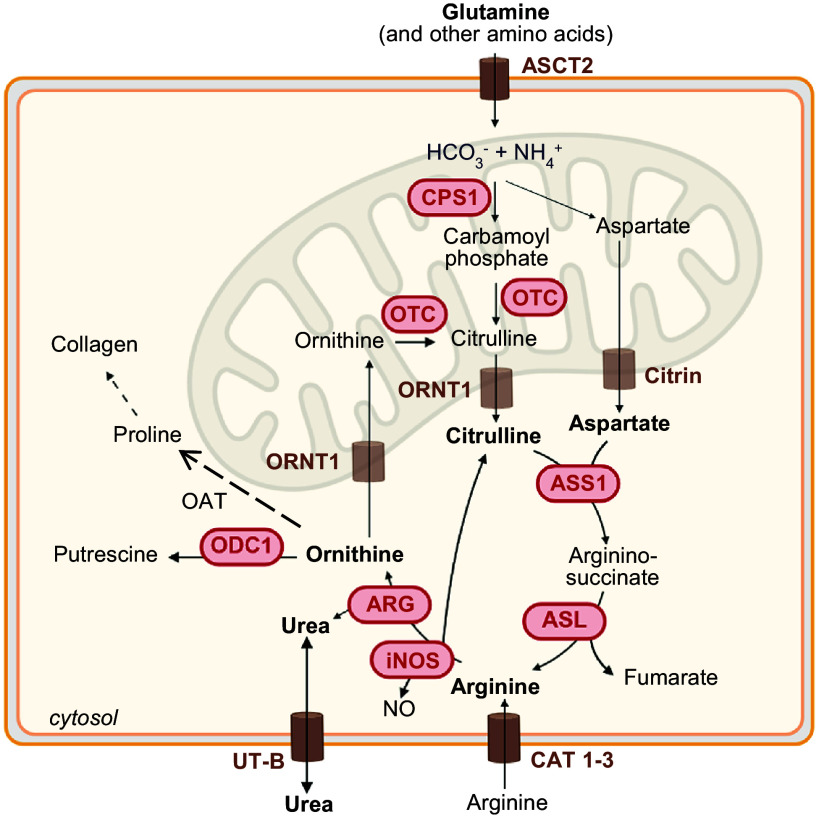
Schematic representation of the urea cycle with its main enzymes and key metabolites. ASCT2 (*SLC1A5*): Alanine, Serine, Cysteine Transporter 2; ASS1: argininosuccinate synthase; ARG: arginase; ASL: argininosuccinate lyase; AST: aspartate aminotransferase; CAT: cationic amino acid transporter; CPS1: carbamoylphosphate synthase 1; Citrin (*SLC25A13*); OAT: ornithine aminotransferase; ODC: ornithine decarboxylase; ORNT1: ornithine transporter (*SLC25A15*); OTC: ornithine transcarbamoylase; iNOS: inflammatory nitric oxide synthase; NO: nitric oxide; UT-B: type B urea transporter. Created in https://BioRender.com. Figure 2. long description.

The main physiological function of the UC is to eliminate toxic ammonium, which is produced during the catabolism of amino acids (Ref. 16). This is achieved through the generation of urea, which is finally excreted via the urine. In the human body, the UC mainly takes place in the liver. Although UC activity is much lower in peripheral tissues, including the prostate, several enzymes of the UC are involved in synthesis of nitric oxide (NO), polyamines, proline and glutamate in non-hepatic tissues (Ref. 17). The key metabolites involved in the UC are glutamine, ornithine, citrulline, aspartate, arginine, and urea (Figure 2). It comprises five catalytic enzymes carbamoyl phosphate synthase 1 (CPS1), ornithine transcarbamylase (OTC), argininosuccinate synthase 1 (ASS1), argininosuccinate lyase (ASL) and arginase (ARG), two amino acid transporters, citrin (*SLC25A13*) and ornithine transporter 1 (ORNT1, *SLC25A15*) and one cofactor producing enzyme (N-acetylglutamine synthase, NAGS) (Ref. 18). The first step of the UC takes place in the mitochondria and involves the production of carbamoyl phosphate by combining ammonium ion (NH_4_^+^) and bicarbonate ion (HCO_3_^−^) through the CPS1. The OTC then transforms carbamoyl phosphate and ornithine into citrulline, which is transported into the cytosol through the ornithine transporter ORNT1 (Ref. 19). There, citrulline is converted with aspartate into argininosuccinate by ASS1 (Ref. 16), which is further cleaved into arginine and fumarate through ASL. Aspartate, which is produced during protein breakdown or as an intermediate product of the TCA cycle, is obtained from the mitochondria through citrin (Ref. 20).

In the final step of the UC, arginine is cleaved into urea and ornithine through ARG. Whereas urea is excreted, ornithine can be either converted into putrescine by ornithine decarboxylase 1 (ODC1) and serve as a precursor for the synthesis of polyamines (Ref. 19) or it is converted to proline by the enzyme ornithine aminotransferase to further synthesise collagen (Ref. 21), or it is shuttled into the mitochondria via ORNT1 to re-start the UC (Ref. 22). ARG is one of the central enzymes in the UC, which exists as two isoforms, the cytoplasmic ARG1, which is mainly found in the liver, and the mitochondrial ARG2, which is broadly expressed among different organs (Ref. 23). If arginine is not converted to urea and ornithine, it can also serve as a substrate of inflammatory nitric oxide synthase (iNOS), which produces citrulline and NO, thereby contributing to inflammation and immune response (Refs 24–26).

Due to its main function of eliminating toxic byproducts from amino acid catabolism, the UC is also strongly influenced by various cell membrane receptors which mediate the uptake of amino acids into the cells (Ref. 16). Most of them belong to the solute carrier family (SLC) and have been recently reviewed (Ref. 27). Worth mentioning with respect to its importance in the UC is the alanine/serine/cysteine transmembrane receptor 2 (ASCT2, encoded by *SLC1A5*) which is the main transmembrane receptor for glutamine (Ref. 28). Glutamine is the most abundant and the most rapidly consumed amino acid in human plasma which plays a crucial role in replenishing the TCA cycle in particular under glucose-deprived conditions (Refs 29, 30). In addition, the large amino acid transporters LAT1 (*SLC7A5*) and LAT2 (*SLC7A8*) are critical transmembrane receptors which mediate the uptake of the essential amino acids isoleucine, leucine and valine (Refs 31–33). A critical role also plays cationic amino acid transporter (CAT) proteins 1–3 (*SLC7A1–3*) which mediate the uptake of arginine into the cell through the diet (Refs 30, 34). Another intriguing transmembrane receptor is the type B urea transporter (UT-B) which is encoded by *SLC14A1* and which has been previously associated with PCa (Ref. 35). UT-B facilitates the gradient-dependent passive transport of urea across the cell membrane and thereby regulates its intracellular homeostasis (Ref. 36).

## Dysregulation of the urea cycle in prostate cancer cells

**Figure 3.**
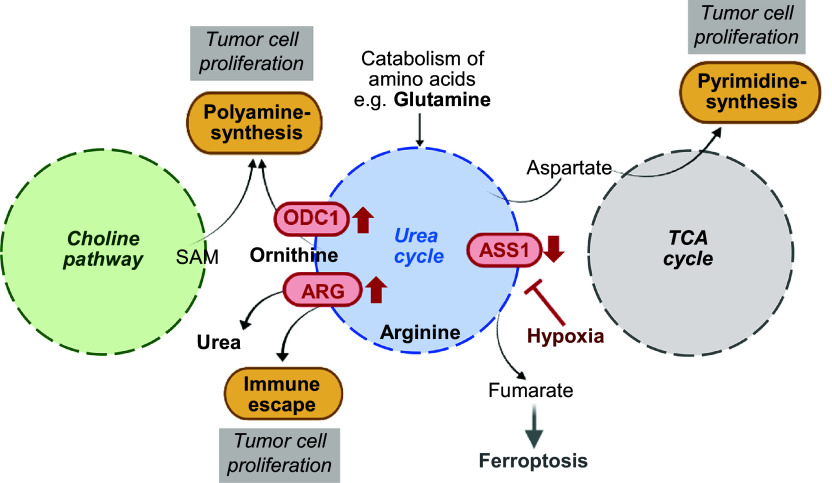
Schematic image showing the crosstalk between the urea cycle, the choline pathway, the TCA cycle, and polyamine synthesis with some of the key metabolites promoting tumour cell proliferation through stimulating polyamine and pyrimidine synthesis and immune escape. SAM: S-adenosylmethionine; ASS1: argininosuccinate synthase; ARG: arginase; ODC: ornithine decarboxylase; TCA: tricarboxylic acid. Created in https://BioRender.com. Figure 3. long description.

There is strong evidence that the UC undergoes major reconfiguration which diverts byproducts from amino acid catabolism towards anabolic pathways to support tumour growth and progression (Figure 3). In particular, UC dysregulation promotes polyamine and pyrimidine synthesis (Refs 37–39) and modulates the immune response in the TME (Refs 24–26, 40), thereby contributing to cell proliferation.

Several studies demonstrated dysregulated expression of UC enzymes in PCa cells (Refs 41, 42) as well as in prostate tumour tissue samples (Ref. 39). In their study, Lee and coworkers showed reduced expression of ASL, ORNT1, ASS1 and OTC which was associated with increased expression of proliferating cell nuclear antigen (Ref. 39). Diminished expression of ASS1 in PCa tissue was also found in other previous studies (Refs 43, 44). Corresponding with these data, benign RWPE-1 cells exhibited high ASS1 expression, whereas PC-3 and CWR22Rv1 PCa cells had low to no expression of ASS1 (Ref. 44). Of note, a multi-omics study comprising various PCa cells and sphere-derived PCa stem cells recently reported on elevated expression of ASS1 together with increased arginine levels, which were particularly found in the cancer stem cell population (Ref. 45). It is assumed that low expression of ASS1 renders aspartate available for pyrimidine synthesis (Ref. 39) and also fuels the TCA cycle, where it is required to produce oxalacetate (Ref. 16). In addition, dysregulated expression of ASS1 and ASL alters the availability of fumarate, thereby controlling the cells’ sensitivity to ferroptosis, an iron-dependent lipid peroxidation driven form of programmed cell death (Ref. 46) (Figure 3). Of note, ASS1 downregulation is controlled by hypoxia-inducible factor 1 subunit α (HIF1α) (Ref. 42) which promotes an oxygen-responsive transcriptional reprogramming through the FOXA1-AR axis, resulting in PCa cell proliferation and metastatic progression (Refs 47–49). Importantly, *de novo* synthesis of arginine is low in cells with a low ASS1 expression, rendering arginine a conditionally essential amino acid, which is exceedingly taken up through the diet (Figure 2). This phenomenon is known as arginine auxotrophy (Ref. 50).

Arginine is one of the key precursor molecules in the synthesis of polyamines, which drive tumour growth and progression. The central enzymes ARG1 and ARG2 were both detected in PCa cell lines with ARG2 being predominantly expressed in AR-positive LNCaP and 22Rv1 cells and ARG1 in AR-negative PC-3 and DU145 cells (Ref. 40). Substantial expression of ARG2 was also shown in human PCa tissue samples (Refs 40, 51). Elevated levels of arginine and glutamate together with increased expression of ARG1 were detected in a castration-resistant LNCaP derivative cell line compared to parental androgen-sensitive LNCaP (Ref. 52). ARG also plays a central role in the production of NO (Figure 2), thereby contributing to cancer initiation, invasion, metastasis and angiogenesis (Ref. 30). High levels of iNOS, an enzyme with a critical role in NO production, have been recently detected in advanced PCa (Ref. 53), indicating that ARGs do not only promote polyamine synthesis but can also contribute to an immunosuppressive tumour environment (Refs 24–26, 40). Of note, ARG1 is the primary isoform expressed by immune cells, particularly tumour-associated myeloid-derived suppressor cells (MDSCs) and tumour-associated macrophages (TAMs), where it contributes to immunosuppression by depleting L-arginine, thus hindering T-cell activity (Ref. 40).

One of the final enzymes in the UC for polyamine synthesis is ODC1, which catalyses the conversion of ornithine into putrescine. A previous study demonstrated that exogenous overexpression of ODC1 renders non-tumourigenic prostate epithelial RWPE-1 cells tumourigenic through mutual activation of the androgen receptor (AR) signalling axis (Ref. 54). High ODC1 expression was also found in pre-cancerous high-grade prostatic intraepithelial neoplasia (PIN) lesions, which was accompanied by elevated ratios of the polyamines spermidine:spermine (Ref. 55). Significant alterations in spermine synthesis and energy metabolism were also found associated with PCa recurrence after prostatectomy (Refs 56, 57). Of note, a recent study discovered that the urea transporter UT-B (*SLC14A1)*, which is used for the extracellular transport of urea, was depleted in PCa tissue, suggesting that ornithine production and polyamine synthesis are promoted instead (Refs 58, 59). Diminished expression of *SLC14A1* was recently attributed to hypermethylation of the *SLC14A1* promoter regions and related to poor prognosis of PCa patients (Refs 35, 45). In their study, Luo and coworkers showed that UC dysregulations with elevated levels of arginine and proline were particularly associated with PCa stemness which was mediated via the JAK2/STAT3 signalling pathway (Ref. 45). Of note, inhibiting *SLC14A1* was associated with suppression of CDK1/CCNB1 pathway and mammalian target of rapamycin (mTOR)/MMP-9 signalling resulting in inhibition of PCa cell proliferation (Ref. 35). Corresponding with these data, activation of mTORC1 was shown to enhance the expression of the enzyme S-adenosylmethionine decarboxylase 1 (AMD1). This enzyme decarboxylates S-adenosylmethionine (SAM) from the choline pathway, a rate-limiting step in polyamine synthesis (Refs 60, 61). There is increasing evidence that cancer cells also exhibit an active polyamine transport mechanism which tightly regulates the maintenance of a continually high intracellular polyamine pool which is required for proliferation, although the exact mechanism of polyamine uptake into the cells is still a matter of investigations (Refs 62, 63).

Dysregulation of the UC has also been associated with changes in the genetic landscape of PCa cells such as loss of phosphatase and tensin homolog (PTEN) or c-MYC amplification (Ref. 30). Increased levels of ornithine and urea were detected in the tissue of PTEN knockout mice compared to wild type prostate (Ref. 64). Arginine metabolism has been previously associated with constitutive expression of the proto-oncogene c-MYC (Refs 65, 66). MYC has also been shown to stimulate polyamine synthesis and uptake through the polyamine transport system (Ref. 67). In addition, MYC, as well as KRAS and PI3K/AKT/mTOR pathway supports the availability of glutamine (Ref. 68). KRAS, for instance, was recently shown to influence glutamine availability within the cells by regulating glutamate dehydrogenase and glutamic oxalo-acetic transaminase (Ref. 30).

## The role of the androgen signalling cascade in urea cycle dysregulation in prostate cancer cells

Androgens with the AR as the central molecule of the androgen signalling cascade are known to significantly influence the metabolic activity of PCa cells (Refs 69, 70). Their particular action on lipid metabolism has been adequately reviewed previously (Refs 71–73). Correspondingly, ADT induces not only apoptosis and cell death of PCa cells but also an overall depression in energy metabolism by significantly affecting glycolysis and lipid metabolism (Ref. 15). Androgens also have a significant impact on the regulation of UC enzymes. In androgen-sensitive PCa cells, ARG2 expression and activity are largely dependent on androgens, thereby promoting proliferation and immune suppression in hormone-sensitive stages (Ref. 40). In addition, ARG activity is changed during hormone therapy (Ref. 19). Integrative analysis of transcriptomic and metabolomic data showed elevated levels of arginine and glutamate together with increased expression of ARG1 in a castration-resistant LNCaP derivative cell line compared to parental androgen-sensitive LNCaP (Ref. 52), suggesting a role in tumour growth and progression. This is likely accomplished through the activation of oncogenic signalling pathways such as phosphatidylinositol-3 kinase (PI3K)/AKT/mTOR, signal transducer and activator of transcription 3 (STAT3), and mitogen-activated protein kinase (Ref. 22). In particular, arginine was found to directly activate mammalian target of rapamycin complex 1 (mTORC1), a nutrient-sensing kinase, thereby leading to an upregulation of oxidative phosphorylation genes and stimulating cell proliferation and survival of PCa cells (Ref. 74). The AR also regulates the expression of ARG2, ODC1, and AMD1. High expression levels of these UC enzymes were found in PCa cells with high AR expression. In addition, their expression was reduced by AR knockdown (Ref. 75). In addition, the expression of SLC14A1 was shown to be affected by androgen deprivation in PCa tissue from patients 3 days after surgical castration (Ref. 76).

## The urea cycle as a potential target to treat prostate cancer and therapy-related side effects

Polyamine synthesis is an important oncogenic pathway in PCa, which is closely linked to the UC on the one hand and to the choline pathway on the other. Moreover, the UC is cross-linked with the TCA cycle (Refs 39, 60) (Figure 3). Targeting the availability of key UC metabolites, such as arginine, ornithine and glutamate, may therefore be an effective way to inhibit tumour growth. This may be achieved by directly inhibiting the respective UC enzymes or by intervening with oncogenic signalling pathways which are linked to the UC. Worth mentioning in this regard is the combined use of the AR inhibitor enzalutamide with the ferroptosis inducer RSL3 which was shown to suppress LNCaP and 22Rv1 PCa cell growth *in vitro* as well as *in vivo* (Ref. 77). Furthermore, inhibiting dihydroorotate dehydrogenase (DHODH), which drives *de novo* pyrimidine synthesis in the presence of aspartate, through the selective DHODH inhibitor orludodstat was shown to inhibit AR-positive and AR-negative PCa cell proliferation (Ref. 78).

### Targeting arginine

Deprivation of arginine has gained renewed interest as a potential therapeutic strategy for arginine auxotrophic tumour cells with low or lost expression of ASS1 (Ref. 25). Arginine starvation was shown to impair mitochondrial function, polyamine synthesis and glycolysis caused by transcriptional suppression of metabolic genes in various PCa cell lines *in vitro* as well as *in vivo* (Ref. 79). In a recent study, it was shown that arginine modulates mitochondrial oxidative phosphorylation in PCa cells through epigenetic regulation of the transcriptional enhancer factor-3 (TEF-3), which is encoded by the TEA Domain Transcription Factor 4 (TEAD-4) gene. Of note, benign prostate RWPE-1 cells, which express high levels of ASS1, were not affected by arginine starvation (Ref. 74). Another study demonstrated that silencing ARG1 and ARG2 inhibited LNCaP PCa cell proliferation (Ref. 40). The most common approach to intervene with the UC is through arginine-degrading enzymes. Recombinant human arginase (rhArg), for instance, causes significant cytotoxicity in LNCaP, DU-145 and PC-3 PCa cells *in vitro* through inhibition of mTOR (Ref. 80). The most frequently investigated drug is arginine deiminase (ADI), a microbial enzyme that converts arginine into citrulline and ammonia to reduce intracellular arginine levels, thereby exhibiting anti-proliferative, antioxidant and anti-inflammatory properties. The use of this drug in the clinic is, however, limited due to its short circulating half-life in plasma and its high immunogenicity (Ref. 81). Therefore, improved drugs, which were chemically modified with polyethylene glycol (PEG), such as PEGylated ADI (ADI-PEG 20) have been developed. In CWR22Rv1 and PC-3 PCa cells, ADI-PEG 20 induced growth inhibition through autophagy *in vitro* and *in vivo.* LNCaP cells, which exhibit high expression levels of ASS1, by contrast, did not respond to the drug (Ref. 44). Twenty years ago, a phase I clinical trial showed promising effects of a combined arginine deprivation therapy with ADI-PEG 20 in combination with docetaxel in patients with advanced PCa (ClinicalTrials.gov, NCT01497925) (Ref. 82). More recently, a similar drug, PEG-BCT-100, a PEGylated recombinant human arginase 1, recently showed anticancer activity in PCa patients (Ref. 83). A phase I/II study of ADI-PEG 20 with carboplatin and cabazitaxel has been initiated for men with aggressive variant PCa to find the best drug concentration for the treatment of PCa with aberrant expression of the tumour suppressor proteins TP53, retinoblastoma (RB1) or PTEN. According to the latest update, the study has been suspended, which means it has been stopped early but may be re-started (ClinicalTrials.gov, NCT06085729) (Table 1). In addition, an open-label, phase I dose-escalation study was conducted to investigate the pharmacological and safety aspects of PEGylated human arginase PEG-BCT-100 in castration-resistant prostate cancer (CRPC) patients. To date, no results from that study have been published for PCa patients (ClinicalTrials.gov, NCT02285101). Of note, there are still efforts to further improve drug pharmacology, which was achieved by coupling human arginase with PEG and Mn^2+^ and Co^2+^ [HuArgI (Co)-PEG5000], showing growth inhibition of DU-145 and PC-3 cells within 120 hours of treatment *in vitro* (Ref. 84). A phase 2 study investigated oral L-arginine versus placebo with or without PDE-5 inhibitors in men with erectile dysfunction who had not biochemically relapsed after primary radiation therapy for locally confined PCa. The primary objective of the study was to find the best dose of L-arginine and, secondarily, to evaluate therapy-related toxicity. The study revealed that the L-arginine-based nutritional supplement had few side effects on the one hand but did not show any significant benefit in terms of improving erectile function in PCa patients who had previously undergone radiation therapy on the other (ClinicalTrials.gov, NCT01105130).

**Table 1.** Current clinical trials to investigate UC targeting drugs in prostate cancer Table 1. long description.

Patient cohort/Purpose of the study	Drug	Target	Dysregulation in PCa	Trial	Status/Result	Study URL
mCRPC patients/drug safety and toxicity	ADI-PEG 20 + Docetaxel	Arginine	Elevated (Refs 45, 52)	Phase 1	Completed/promising clinical activity in mCRPC patients (Ref. 82)	https://clinicaltrials.gov/study/NCT01497925
PCa patients treated with radiation therapy/effect on erectile function	Oral L-Arginine	–	–	Phase 2	Completed/no significant benefit on erectile function in PCa patients	https://clinicaltrials.gov/study/NCT01105130
CRPC patients	PEG-BCT–100	ARG	Elevated (Refs 30, 40, 51, 52 )	Phase 1	Completed/no results posted	https://clinicaltrials.gov/study/NCT02285101
PCa patients under ADT/effect on muscle loss	ADT + HMB + Arginine + Glutamine versus ADT	–	–	Phase 2	Completed/ positive trend in preserving muscle function in men undergoing ADT	https://clinicaltrials.gov/study/NCT01607879
Men at high risk for PCa/chemoprevention	DMFO versus Placebo	ODC	Elevated (Ref. 55)	RCT Phase 2b	Completed/decrease in prostate putrescine levels and reduced prostate volume (Ref. 94)	https://clinicaltrials.gov/study/NCT00006101
mCRPC patients with progressive disease after abiraterone treatment/treatment effectiveness	Standard of care ADT + repeat sequential DFMO + high-dose testosterone in sequence with enzalutamide (Single Arm)	ODC	Elevated (Ref. 55)	Phase 2	Recruiting/no results posted	https://clinicaltrials.gov/study/NCT06059118
Patients with localised PCa undergoing brachytherapy or radical prostatectomy/drug effectiveness	Neoadjuvant DFMO + bicalutamide versus DFMO versus bicalutamide versus no neoadjuvant therapy	ODC	Elevated (Ref. 55)	RCT Phase 2	Completed/no results posted	https://clinicaltrials.gov/study/NCT00086736
mCRPC patients without mutations in HRR genes (incl. BRCA1/2 or ATM) who have progressed after docetaxel and either abiraterone or enzalutamide	Telaglenastat (CB–839) + Talazoparib (sequential assignment)	GLS1	Elevated (Ref. 104)	Phase 2	Unkown Status^a^/no results posted	https://clinicaltrials.gov/study/NCT04824937
Aggressive variant of mCRPC, e.g. neuroendocrine PCa or exclusive visceral metastasis or known loss or mutation in at least 2 of TP53, RB1 and PTEN, etc.	ADI-PEG 20 + Carboplatin + Cabazitaxel	Arginine	Elevated (Ref. 55)	Phase 1/2	Suspended/no results posted	https://clinicaltrials.gov/study/NCT06085729

Abbreviations: ADI-PEG: PEGylated arginine deiminase; ADT: androgen deprivation therapy; ARG: arginase; CRPC: castration-resistant prostate cancer; DMFO: Difluoromethylornithine; GLS: glutaminase; HMB: hydroxymethylbutyrate; HRR: homologous recombination repair; mCRPC: metastatic CRPC; mHSPC: metastatic hormone-sensitive prostate cancer; NCT: National Clinical Trial; ODC: ornithine decarboxylase; PCa: prostate cancer; PEG-BCT: PEGylated Recombinant Human Arginase I; RCT: randomised controlled trial.

a Study has passed its completion date and status has not been verified in more than 2 years at clinicaltrials.gov.

Another opportunity to intervene with hyper-activation of the UC is by blocking arginine uptake through inhibition of CATs with verapamil, a calcium channel blocker used to treat hypertension. More than 20 years ago, LNCaP cells were shown to be inhibited by verapamil *in vitro* (Ref. 85). Clinical trials, however, revealed conflicting results regarding a potential use of verapamil or other calcium channel blockers in the treatment of PCa. A recent meta-analysis of published data revealed that PCa patients who take verapamil, or any other calcium channel blocker even have a higher risk of PCa (Ref. 86). Calcium channel blockers like verapamil and also other vasodilators have been spotlighted in the use as anticancer drugs in the past. Their clinical use, however, should be carefully estimated with regard to potential off-target effects which may vary depending on the specific drug and the type of cancer (Ref. 87). Verapamil, for instance, has been shown to influence glucose metabolism through interaction with thioredoxin-interacting protein expression (Ref. 88). In addition, verapamil was shown to interact with potassium channels (Ref. 89) and adrenergic receptors (Ref. 90) and is therefore not considered as a selective CAT inhibitor.

Arginine and glutamine, when combined with β-hydroxy-β-methylbutyrate (HMB), play a key role in supporting protein synthesis and preserving muscle mass. Clinical trials have shown that this combination improves strength, functionality, and fat-free mass in elderly adults and cancer patients with muscle loss (Refs 91, 92). Thus, a phase 2 study in PCa patients starting ADT investigated the protective effects of arginine and glutamine with focus on therapy-related muscle decline by enhancing protein synthesis alongside HMB’s protective effects. Preliminary results indicate that supplementation with HMB, arginine and glutamine is well tolerated and shows a positive trend in preserving muscle function in men undergoing ADT (ClinicalTrials.gov, NCT01607879).

### Targeting ornithine

Polyamine synthesis can be efficiently blocked by targeting ODC1. In transgenic adenocarcinoma mouse prostate (TRAMP) mice, orally applied α-difluoromethylornithine (DFMO), an inhibitor of ODC1, resulted in a significant decrease in prostate volume and weight as well as in reduced ODC enzyme activity and metastasis (Ref. 93). In a randomised placebo-controlled trial with PCa patients, DFMO decreased putrescine levels in the prostate by 60.8% and also delayed prostate growth compared to the placebo arm (Clinicaltrials.gov/study/NCT00006101) (Ref. 94). In a currently recruiting clinical trial, the effect of DFMO is tested in combination with high-dose testosterone and enzalutamide in patients with metastatic CRPC who are progressive after treatment with abiraterone (ClinicalTrials.gov, NCT06059118). Another randomised, placebo-controlled phase II trial tested DFMO, bicalutamide or their combination as short-term neoadjuvant therapy in men with localised prostate cancer prior to surgery or brachytherapy to determine whether these treatments, alone or together, reduce tumour-promoting polyamines (spermine, spermidine and putrescine), alter key biomarkers of proliferation and apoptosis and remain tolerable. Although the randomised controlled trial (RCT) was finished in 2003, no study results were published yet (ClinicalTrials.gov, NCT00086736) (Table 1). Of note, recent studies have demonstrated that cancer cells may compensate inhibition of polyamine synthesis through DFMO by increased polyamine uptake through the polyamine transport system. Recent studies therefore explored the effects of combined inhibition of polyamine synthesis inhibitors like DFMO with polyamine transport inhibitors (Refs 62, 67). *In vitro*, Devens et al. showed that PCa cells (LNCaP, PC-3, DU145) were inhibited DFMO in combination with ORI 1202, a novel inhibitor of polyamine uptake into the cell. Together, these drugs induced a reduction of intracellular putrescine and spermidine levels in PCa cells, resulting in reduced tumour cell growth (Ref. 38). Noteworthy, the dysregulated polyamine transporter expression may also be used for targeted delivery of therapeutic or diagnostic substances. In a PC-3 xenograft model, linking radiolabeled probes to spermine resulted in improved uptake of the probe via the polyamine transport system, suggesting a promising strategy for therapy as well as diagnostic imaging (Ref. 67). Unfortunately, there are no studies available so far which would confirm a clinical application of this approach in PC patients.

An interesting drug, which was shown to reduce the expression of UC enzymes such as CPS1, ARG1, OTC and ODC1, is metformin, a drug frequently prescribed for the treatment of type 2 diabetes (Ref. 95). In two completed retrospective clinical studies, metformin was shown to prolong overall survival in patients with mPCa (Ref. 96). There have been several clinical trials so far, however, with somehow inclusive results whether or not PCa patients might benefit from metformin. A Phase II randomised study with overweight or obese PCa patients (BIMET-1) that was undertaken to investigate the effect of metformin in combination with the antiandrogen bicalutamide on biochemical recurrence was stopped early due to a predicted response failure (Ref. 97). A recent study reported that response to metformin is likely dependent on NKX3.1 expression level. Among its diverse cellular functions, this prostate-specific homeobox transcription factor protects mitochondria from oxidative stress (Ref. 98). In fact, PCa patients with low NKX3.1 had a longer delay of disease progression after metformin treatment compared to patients with high NKX3.1 levels (Ref. 99).

A potentially interesting strategy is not directly targeting ornithine but the enzyme pyrroline-5-carboxylic acid which catalyses the formation of proline from ornithine (Ref. 21). Knocking-out this enzyme with short hairpin RNAs inhibited DU145 cells *in vitro* and *in vivo* (Ref. 45). Similarly, intervening with mTORC1-dependent regulation of AMD1 stability with the mTORC1 inhibitor everolimus resulted in a decrease in AMD1 expression in biopsy tissue from PCa patients (Ref. 61).

### Targeting glutamine

Increasing evidence suggests that PCa metabolism is significantly driven by glutamine, in particular in advanced and castration-resistant forms (Ref. 72). *In vitro* culture of PCa cells in glutamine-deprived medium induces growth suppression of androgen-independent DU-145 and PC-3 cells (Ref. 100) and of androgen-sensitive LNCaP cells through inhibition of mTORC1 (Ref. 101). Glutamine enters the cells mainly via ASCT2 and is catabolised into glutamate via glutaminase 1 (GLS1). Importantly, GLS1 is frequently upregulated in PCa cells such as LNCaP and PC-3 cells and is also highly expressed in PCa tissue (Ref. 102). In line with these data, glutamine levels were found to be elevated in plasma of PCa patients compared to benign volunteers (Ref. 103). A recent study demonstrated improved inhibition of advanced PCa cell growth by combined targeting of arginine and glutamine metabolism through knocking down CAD and treatment with CB-839, a selective GLS1 inhibitor (Ref. 104), suggesting that targeting arginine and glutamine metabolism together may be the most effective strategy to inhibit prostate tumour growth.

For clinical applications, disrupting glutamine activity is preferentially performed with the help of GLS1 inhibitors which showed high safety and tolerability in clinical studies (reviewed by (Ref. 105)). Telaglenastat (CB-839), for example, is currently investigated in a phase II clinical trial in combination with Poly-ADP-ribose polymerase inhibitors for metastatic CRPC. To date no results from this study have been published (ClinicalTrials.gov, NCT04824937) (Table 1). Interference with glutamine metabolism may alternatively be achieved by targeting SLC1A5/ASCT2 with short hairpin RNAs. This strategy was shown to inhibit PC-3 xenograft growth and invasiveness *in vivo* (Ref. 106). Of note, recent studies have shown that suppressing GLS1 alone may result in the elevation of pyrimidine synthesis in PCa cells. This compensation can be abolished through simultaneous blockade of GLS1 and dihydroorotase (Ref. 104).

With all these new approaches to intervene with the metabolic reprogramming of PCa cells, we always need to carefully consider the known heterogeneity of PCa tissue which may hamper drug effectiveness in patients. Increasing numbers of single-cell and spatial transcriptomic studies discovered spatially variable expression of metabolic genes across human prostates (Refs 59, 107). In their study, Wang et al., for example, found fumarate dehydratase and succinate dehydrogenase selectively depleted in PCa cells (Ref. 59). The identification of such cell-type-selective metabolic vulnerabilities is important with regard to drug effectiveness. In addition, spatial transcriptomics may also help to discover new targets which might have been missed by models using bulk sequencing and provides important information on the metabolic activity within the tumour microenvironment (TME) and its crosstalk with the tumour cells.

### Targeting the UC within the tumour microenvironment

The prostate TME plays an intriguing role in the metabolic reprogramming of various pathways, including the UC (Ref. 27). The various different cell types present in the TME, including adaptive immune cells, TAMs, cancer-associated fibroblasts (CAFs), and myeloid-derived suppressor cells (MDSCs), are important modulators of the tumour cells’ metabolic phenotype. This fact must be taken into account when developing new effective therapies that target tumour cell metabolism (Ref. 59). As mentioned above, arginine can also regulate immune cell activity through the production of NO from arginine. This is considered the main communicating metabolite between tumour cells and TAMs (Refs 20, 108, 109). TAMs are the most prominent cellular components in the TME and are usually classified into classically activated pro-inflammatory M1-type and alternatively activated anti-inflammatory M2-type macrophages, respectively (Ref. 110). Findings from studies on TAMs with an M2-like phenotype indicate that iNOS/ARG balance within macrophages is relevant for tumour progression (Ref. 111). In a TRAMP mouse, PCa model irradiation enhanced the expression of ARG-1 and iNOS in M2 phenotype TAMs, suggesting impaired immune response as a possible mechanism for treatment resistance (Ref. 112). Corresponding with this, the presence of M2 macrophages in PCa tissue correlated with tumour progression (Ref. 113) and poor prognosis (Ref. 53). ARG was also shown to regulate the function of T-cells in the TME which is often due to downregulation of ASS1 resulting in anti-tumour immune escape (Refs 114, 115). These data suggest that targeting the UC, in particular, ARG2, might be a promising puzzle in immunotherapeutic strategies for PCa patients (Ref. 116). Most of the published studies focused on improving the efficacy of immune therapy for PCa patients by reversal of immunosuppressive signals. This is particularly important in the treatment of PCa which is considered an immune-resistant ‘cold’ tumour where immunotherapy often has limited success (Ref. 117). Antagonists for ARG1 and iNOS, for instance, were shown to restore T-cell mediated cytotoxicity in PCa (Refs 19, 118, 119). Importantly, it has been hypothesised that these drugs may be effective in androgen-dependent PCa where ARG2 expression is high but would be less potent in androgen-independent PCa with decreased ARG2 levels (Refs 19, 40, 44). A novel approach to enhance the effectiveness of immunotherapy in PCa is the use of chimeric antigen receptor T (CAR-T) cells (Ref. 120). Of note, reduced availability of arginine together with low expression of arginine synthesis enzymes has been shown to hamper the efficacy of CAR-T cells in haematological and solid malignancies (Ref. 109).

Besides TAMs, also CAFs are known to play an intriguing role in modulating the tumour cells’ metabolism through a paracrine reciprocal feedback loop between tumour, stromal and immune cells which finally results in sustaining tumour growth and progression towards therapy resistance as recently reviewed (Refs 121, 122). CAFs are known to interact with PCa cell metabolism most likely by providing lactate and glutamate (Ref. 123). A recent study showed that ammonium stimulates metastatic PCa cells whereas it slows the growth of fibroblasts (Ref. 124). UC dysregulations in CAFs have been previously reported for various other cancer types (Ref. 115) but are limited for PCa so far.

## UC metabolites for diagnostic purposes in prostate cancer

The detection and monitoring of PCa is still predominantly confined to measuring prostate-specific antigen (PSA) in the blood, despite its lack of specificity and the risk of over diagnosis and potential overtreatment (Ref. 125). For this reason, efforts to develop more precise diagnostic and prognostic markers are being intensively continued. The frequently reported dysregulated UC metabolites also have been suggested for this purpose. Markin et al., for example, reported on a significant increase in urea, ornithine and glutamine in the urine of PCa patients (*n* = 27) compared to tumour-free controls (*n* = 36), and patients with PIN (Ref. 126). The same group of researchers also reported on plasma sarcosine levels as marker to distinguish prostate PIN and benign prostatic hyperplasia (BPH) from PCa patients, respectively (Ref. 127). A similar study by Wang et al. revealed a sarcosine/creatinine ratio as potential diagnostic indicator of PCa in patients with a PSA lower than 10 ng/ml (Ref. 128). Kumar et al. measured increased levels of sarcosine in serum of PCa patients compared to benign patients with BPH (Ref. 129). Schmidt et al. reported on an increased risk of PCa when citrulline serum levels were low (Ref. 130). Arginine was found elevated in PCa tissue compared to benign controls (Ref. 131). Another study showed that urine samples as well as tissue from PCa patients exhibited higher levels of arginine compared to patients with BPH (Ref. 132). Corresponding with these data, we identified a 22-metabolite panel in sera of PCa patients (*n* = 750) that was significantly different from benign patients (*n* = 429) with elevated PSA. Among these 22 significantly altered metabolites were ornithine, urea, and glutamine (Ref. 133). Uric acid and urea were also identified as potential diagnostic markers for PCa by Heger et al. (Ref. 134). High ornithine but low arginine levels were detected in plasma of 78 PCa patients with elevated PSA levels (4–10 ng/ml) by Selvi and coworkers (Ref. 135). Similarly, glutamine has been considered as a biomarker to discriminate PCa from BPH. Increased glutamine levels were detected in plasma (Ref. 136) and in urine of PCa compared to BPH patients (Ref. 137). Despite these discriminatory differences between benign and malignant prostate diseases, none of these metabolites has found its way into clinical diagnostic practice so far. At least partly, this may be attributable to problems with standardisation and comparability of the respective analytical methods (Ref. 138).

## Expert and topical summary

The current research status clearly shows that reprogramming of the UC plays an important role in PCa. An increasing number of preclinical but also some early-stage clinical studies provide encouraging data for targeting the UC for improvement of therapy or for overcoming resistance mechanisms. Unfortunately, some clinical trials, which have been listed on clinical.trials.gov, have been completed up to 20 years ago but still lack a publication of results. Anyway, we believe that for an effective use of UC dysregulations for therapeutic purposes in the clinic, we still warrant a better understanding of the complex interactions among the different metabolic pathways and adaptive compensatory strategies of the tumour cells and also their communication with the TME. Further filling the gaps in our knowledge of metabolism and its adaptations in tumours seems a promising route to expand treatment options in the future. Regarding a use in clinical diagnosis, several metabolite panels including UC metabolites were developed to identify PCa. A significant progress in this field of research is most likely dependent on reliable and reproducible results, which can only be achieved by optimisation and standardisation of analytical procedures.

## Long descriptions

### Figure 1. Long description

The diagram consists of two side-by-side panels.

Left Panel: Benign cell.

* At the top, Glucose enters the cell and is converted to Pyruvate, yielding A T P. Pyruvate converts to Lactate or Acetyl C o A.

* Acetyl C o A enters the mitochondrion and combines with Oxaloacetate to form Citrate.

* Inside the mitochondrion, Zinc enters through a channel and inhibits the conversion of Citrate to alpha-K G.

* Consequently, Citrate is exported out of the mitochondrion and out of the cell.

* A small amount of Glutamine enters from the bottom to form alpha-K G, which proceeds through Succinyl-C o A and Oxaloacetate to complete the T C A cycle, yielding A T P.

Right Panel: Tumor cell.

* Glucose, Lactate, Cholesterin, Citrate, Lipids, and Glutamine are highlighted in purple boxes.

* Glucose enters and produces A T P. Pyruvate is converted to Lactate.

* Acetyl C o A is converted to Cholesterin.

* Inside the mitochondrion, there is no Zinc inhibition. Citrate is converted to alpha-K G to drive the T C A cycle, producing a large A T P burst.

* Citrate also exits the mitochondrion to be converted into Lipids via F A S N.

* At the bottom, Glutamine enters the cell and is converted to alpha-K G via G L S to further fuel the T C A cycle.

Navigate back to Figure 1..

### Figure 2. Long description

The diagram depicts the urea cycle across three compartments: extracellular space, cytosol, and the mitochondrion.

* At the top, Glutamine enters the cytosol via the A S C T 2 transporter.

* Inside the mitochondrial matrix, H C O sub 3 minus and N H sub 4 plus are converted by C P S 1 into Carbamoyl phosphate.

* Carbamoyl phosphate combines with Ornithine (facilitated by O T C) to form Citrulline.

* Citrulline exits the mitochondrion via the O R N T 1 transporter into the cytosol.

* In the cytosol, Citrulline and Aspartate (which exited the mitochondrion via Citrin) are converted by A S S 1 into Argininosuccinate.

* A S L then breaks Argininosuccinate into Fumarate and Arginine.

* Arginine has multiple pathways:

- Converted by A R G into Urea and Ornithine.

- Converted by i N O S into N O and Citrulline.

- Transported out of the cell via C A T 1-3.

* Urea is transported out of the cell via U T-B.

* Ornithine can re-enter the mitochondrion via O R N T 1 to continue the cycle, or be converted by O D C 1 into Putrescine, or by O A T into Proline and subsequently Collagen.

Navigate back to Figure 2..

### Figure 3. Long description

The diagram consists of three large dashed circles representing metabolic hubs.

1. On the far left, a green circle labeled Choline pathway outputs S A M. S A M feeds into a yellow box labeled Polyamine-synthesis, which is topped with a gray box labeled Tumor cell proliferation.

2. In the center, a blue circle labeled Urea cycle is the primary hub.

- At the top, an arrow indicates Catabolism of amino acids e.g. Glutamine enters the cycle.

- On the left perimeter of the blue circle, O D C 1 with an upward red arrow and A R G with an upward red arrow are positioned. O D C 1 connects to Ornithine, which also feeds into Polyamine-synthesis. A R G connects to Arginine and leads to two outputs: Urea and a yellow box labeled Immune escape, which is topped with a gray box labeled Tumor cell proliferation.

- On the right perimeter, A S S 1 has a downward red arrow. Hypoxia, shown in red text, is indicated as an inhibitor of A S S 1.

- Below the cycle, Fumarate leads to Ferroptosis.

3. On the right, a gray circle labeled T C A cycle interacts with the Urea cycle.

- Aspartate moves from the T C A cycle into the Urea cycle.

- An arrow from the T C A cycle points upward to a yellow box labeled Pyrimidine-synthesis, which is topped with a gray box labeled Tumor cell proliferation.

Navigate back to Figure 3..

### Table 1. Long description

The table contains seven columns and nine rows of clinical trial data.

* Row 1: m C R P C patients/drug safety. Drug: A D I-P E G 20 plus Docetaxel. Target: Arginine. Dysregulation: Elevated. Trial: Phase 1. Status: Completed/promising clinical activity. U R L: n c t 0 1 4 9 7 9 2 5.

* Row 2: P C a patients treated with radiation/erectile function. Drug: Oral L-Arginine. Target: None. Dysregulation: None. Trial: Phase 2. Status: Completed/no significant benefit. U R L: n c t 0 1 1 0 5 1 3 0.

* Row 3: C R P C patients. Drug: P E G-B C T-100. Target: A R G. Dysregulation: Elevated. Trial: Phase 1. Status: Completed/no results posted. U R L: n c t 0 2 2 8 5 1 0 1.

* Row 4: P C a patients under A D T/muscle loss. Drug: A D T plus H M B plus Arginine plus Glutamine versus A D T. Target: None. Dysregulation: None. Trial: Phase 2. Status: Completed/positive trend. U R L: n c t 0 1 6 0 7 8 7 9.

* Row 5: Men at high risk/chemoprevention. Drug: D F M O versus Placebo. Target: O D C. Dysregulation: Elevated. Trial: R C T Phase 2 b. Status: Completed/decrease in prostate putrescine levels. U R L: n c t 0 0 0 0 6 1 0 1.

* Row 6: m C R P C patients after abiraterone/effectiveness. Drug: A D T plus D F M O plus high-dose testosterone plus enzalutamide. Target: O D C. Dysregulation: Elevated. Trial: Phase 2. Status: Recruiting. U R L: n c t 0 6 0 5 9 1 1 8.

* Row 7: Localised P C a undergoing brachytherapy or prostatectomy. Drug: Neoadjuvant D F M O plus bicalutamide versus D F M O versus bicalutamide versus no therapy. Target: O D C. Dysregulation: Elevated. Trial: R C T Phase 2. Status: Completed/no results. U R L: n c t 0 0 0 8 6 7 3 6.

* Row 8: m C R P C patients without H R R mutations. Drug: Telaglenastat C B-8 3 9 plus Talazoparib. Target: G L S 1. Dysregulation: Elevated. Trial: Phase 2. Status: Unknown. U R L: n c t 0 4 8 2 4 9 3 7.

* Row 9: Aggressive variant m C R P C. Drug: A D I-P E G 20 plus Carboplatin plus Cabazitaxel. Target: Arginine. Dysregulation: Elevated. Trial: Phase 1/2. Status: Suspended. U R L: n c t 0 6 0 8 5 7 2 9.

Navigate back to Table 1..
